# Validating the reliability and validity of the Fear of Cancer Scale (FOCS) in the general population based on modified classical test theory and analyzing its risk factors

**DOI:** 10.1097/MD.0000000000049066

**Published:** 2026-05-29

**Authors:** Li Feng, Yu Chunmei, Wan Fangyun, Wang Wenxing, Wang Xuexing, Zhang Jian, Xu Jun, Gan Lin, Liu Zhijin

**Affiliations:** a Department of Oncology, The Third Affiliated Hospital, Jiangxi Medical College, Nanchang University-The First Hospital of Nanchang, Nanchang, Jiangxi, PR China; b Department of Oncology, Pingxiang People’s Hospital, Pingxiang, Jiangxi, PR China; c Department of Nursing College, Nanchang Medical College, Nanchang, Jiangxi, PR China; d Department of Traditional Chinese Medicine College, Jiangxi University of Chinese Medicine, Nanchang, Jiangxi, PR China; e Department of Oncology, Anning First People’s Hospital affiliated to Kunming University of Science and Technology, Kunming, Yunnan, PR China.

**Keywords:** cancer-related fear, psychometrics, psychosocial oncology, public health, scale validation

## Abstract

To validate the reliability, validity, and applicability of the Fear of Cancer Scale (FOCS) for identifying individuals in the entire Chinese general population at high risk for cancer-related fear, and to analyze the current status and risk factors associated with general population fear of cancer. Using the modified classical test theory, a total of 3963 participants were recruited from the entire Chinese general population from January to October 2024 to validate FOCS. Delphi expert consultation, item analysis and cognitive interview were used to validate the content and face validity of FOCS. Factor analysis and structural equation modeling were used to test the structural validity of FOCS. Cronbach alpha coefficient, Spearman-Brown coefficient were used to test the internal consistency reliability of FOCS. Multiple stepped-linear regression was used to analyze the risk factors of cancer-related fear in the general population. The FOCS of 17 items was divided into 3 dimensions: 8 items for direct fear, 6 items for indirect fear, and 3 items for latent fear. The three-factor correlation model’s FOCS demonstrated strong validity (root mean square error of approximate = 0.042, goodness of fit index = 0.974, AGFI = 0.961, comparative fit index = 0.972, normed fit index = 0.965, incremental fit index = 0.972, tucker-lewis index = 0.962), reliability (Cronbach α coefficient was 0.885, Spearman-Brown coefficient was 0.674) and applicability (average completion time [298.13 ± 191.484 seconds] and effective completion rate of 91.27%). The cancer fear score of 3963 participants was 17 to 85, with *M (P*_25_, *P*_75_) was 55 (48, 63). Sex, age, occupation, and education level were found to be independent risk factors for general population cancer-related fear. The general population commonly experience significant fear of cancer-related issues, and the adjusted FOCS is effective and reliable for early identification and assessment of the level of cancer-related fear, which may provide an effective basis for cancer health education and public health services in the future.

## 1. Introduction

In the past 30 years, the incidence and mortality of cancer have continued to rise globally.^[1]^ Data from the National Cancer Center of China in 2024 showed that the number of new cancer cases and deaths in China reached 4.82 million and 2.57 million respectively, which had become a significant threat to the physical and mental health of the general population as well as socio-economic advancement.^[2–5]^

“Fear” refers to strong psychological depression caused by fear and anxiety in the face of dangerous situations during human social activities.^[3]^ “Cancer-related fear” refers to the negative psychological activities and behavioural manifestations of the general public due to concerns about their own life safety being invaded or threatened by cancer, which includes subjective or objective direct and indirect fears.^[4,5]^ However, studies on cancer-related fear among the general population are rare, especially on a society-wide population scale.

With the development of medicine, although the efficacy and prognosis of cancer treatment have continuously improved, due to a lack of health knowledge, the public’s understanding of cancer is still not objective or comprehensive enough, which leads to a strong fear of cancer among the public.^[6,7]^ The fear of cancer-related conditions among the general population could lead to various stigmatization, humiliation, and discrimination against cancer patients, which is not conducive to the treatment and recovery of cancer patients and seriously damages their quality of life.^[8]^ In addition, the lack of proper guidance and psychological health services may also lead to social panic and public psychological problems.^[9,10]^ Therefore, cancer-related social psychological problems have gradually become a new hot topic of research for scholars worldwide.

Previous studies have shown that the public’s fear of cancer-related stems from cancer stereotypes, the pain of diagnosis and treatment processes, and social isolation. The public’s “cancer peculiar horror” stems from the fact that cancer is a malignant disease that is unpredictable and eventually leads to the patient death.^[11]^ The painful diagnosis and treatment process, high treatment costs, and unavoidable treatment toxicity of cancer are important sources of public fear of cancer-related.^[8,12]^ Because of cancer-related fear, the public always looks at cancer patients with “colored glasses” and consciously reduces communication with them.^[13]^ The emotions of relatives and friends changed from initial concerns to final indifference, and even abandoned cancer patients for fear of their own safety was threatened.^[14]^ In recent years, the heterogeneity of the tumor microenvironment, adverse events related to immune checkpoint inhibitors, and the interaction between chronic diseases and the immune microenvironment have become cutting-edge fields in cancer research, which may become sources of public fear of the unpredictability of cancer.^[15,16]^

However, there is currently no appropriate instrument for evaluating the level of cancer-related fear among the general population. Although the champion’s breast cancer fear scale (CBCFS) could be used to measure women’s fear of breast cancer, and had been proven to have good reliability and validity in many countries, it had limited applicability and could be used only to evaluate the fear of a specific population toward a single cancer.^[17–19]^ In addition, the cancer awareness measure (CAM), which had good reliability and validity, could be used to assess the cognition of the general population on cancer health knowledge, and had been successively revised to be applicable to specific cancers, such as breast cancer, colorectal cancer and hematological malignancies.^[20–22]^ However, the specificity of CAM was limited, and it could be used only to assess public cognition of cancer knowledge. Although the previously developed Fear of Cancer Scale (FOCS) could be used to preliminarily estimate cancer-related fear among college students, its reliability, validity, and applicability in other general populations still need further validation, especially in middle-aged and older adults.^[23]^

Therefore, the purpose of our study was to apply FOCS to the entire Chinese general population, to further test its reliability, validity, and applicability, and to analyze the current status and risk factors for cancer-related fear among the general population.

## 2. Methods

### 2.1. Participants

The general population in public places such as communities, hospitals, schools and factories were used as the research object. Participants from January to February 2024 were selected as cognitive interview objects, and those from February to October 2024 were selected as questionnaire objects (participants from February to April 2024 were used for exploratory factor analysis (EFA), and those from May to October 2024 were used for confirmatory factor analysis).

The inclusion criteria for participants were as follows: had no previous history of malignant tumor disease, good language expression capability and understanding ability, volunteered to participate in the study, and were ≥ 18 years old. The exclusion criteria for participants were as follows: had a history of mental disorders, brain trauma, physical disability or critical illness, or incomplete or untrue questionnaires (including missing answers, multiple answers and the same answer for all items), or had a family history of tumors. Before the formal questionnaire, participants were required to provide written or electronic informed consent and a statement of no primary malignant tumor.

This study had obtained the consent and authorization of the original author of FOCS. Ethical approval for this cross-sectional study was granted by the Ethics Committee of The Third Affiliated Hospital of Nanchang University-The First Hospital of Nanchang (NO.KY2023065) and Anning First People’s Hospital affiliated to Kunming University of Science and Technology (NO.202403702). We promised the participants that the data would only be used for this study, without any commercial use, and that the participants could withdraw from the study at any time. In addition, we confirm that all methods were performed in accordance with the relevant guidelines and regulations.

### 2.2. FOCS pre-investigation review

According to the Delphi expert consultation method,^[24]^ we invited experts in oncology, social psychology, public health, nursing, and Chinese language to assess the content validity, applicability and compatibility of each item in a group meeting format, with appropriate revisions and refinements if necessary. Then, based on the 4-point scoring system, experts were invited to independently score the effectiveness of each item’s content, and the item-level content validity index (I-CVI) and scale-level content validity index (S-CVI) was calculated^[25]^ (From December 2023 to January 2024).

A small portion of general population was invited to participate in cognitive interviews, and a 4-level rating form was used to validate the face validity of the description, content, and significance of each item to ensure that all items could be easily read, understood, and accepted during the formal investigation, and the item-level face validity index (I-FVI) and scale-level face validity index (S-FVI) was calculated^[26]^ (from January to February 2024).

### 2.3. Questionnaire formation and distribution

The questionnaire consisted of 4 parts: The general information of the participants was designed by the researchers according to the needs, including sex, age, residence area, education level, and occupation. The health information and cancer awareness collection form was included. The Myers–Briggs Type Indicator personality types were included, which were used to determine participants’ personality traits (introvert or extrovert).^[27]^ and the reviewed FOCS was included. To test the comprehensibility, acceptability and reality of the questionnaire, 50 to 100 members of the general population were invited to conduct a pre-survey.

The random sampling and snowball sampling methods were used to conduct a questionnaire survey on social groups such as communities, hospitals, schools, and factories through online (electronic questionnaire) and offline (paper questionnaire) synchronization. All questionnaires were independently completed by participants according to their own assumptions, and the completion time of the questionnaire was recorded. To qualify for factor analysis, the ratio between sample and item size was at least 5 to 10, and the common recommendation for the whole Chinese general population sample size was N ≥ 1000.^[28]^ The formal investigation was conducted from February to October 2024.

### 2.4. FOCS validation

Delphi expert consultation, item analysis and cognitive interview were used to verify the face and content validity of FOCS. EFA was used for dimensionality reduction, common factors were extracted by parallel analysis and principal component analysis, and the convergent and discriminant validity of FOCS were examined. Confirmatory factor analysis (CFA) and structural equation modeling were used for verify the construct validity of the FOCS. The Cronbach alpha coefficient, Spearman-Brown coefficient and test-retest reliability were used to verify the reliability of the FOCS. Ordered logistic regression analysis was used to verify the differential item function (DIF) of FOCS in different sex groups. The applicability of the FOCS was verified by the effective completion rate and average completion time.

### 2.5. Risk factors of FOCS among the noncancer population

One-way analysis of variance was used to compare the scores of FOCS among different participants, and multiple stepwise linear regression analysis was used to analyze the independent risk factors of general population FOCS scores.

### 2.6. Statistical analysis

So-Jump 2.0 was used for online questionnaire distribution and recovery. SPSS 25.0 and Excel 2019 were used for statistical description and analysis, Mplus 8.1 was used for parallel analysis, and AMOS 24.0 was used to construct structural equation models. A two-tailed *P* < .05 was considered to indicate statistical significance.

## 3. Results

### 3.1. Participant characteristics

A total of 4342 individuals in the general population agreed to participate, and 3963 effectively completed the entire questionnaire (379 participants with incomplete questionnaires, a history of cancer, or under 18 years of age were excluded). Among the 3963 participants, 2299 were female, and 1664 were male; 3070 were nonmedical staff, and 893 were medical staff (Table 1).

**Table 1:** Participant characteristics and one-way ANOVA of FOCS.

Characteristics		Number (%)	FOCS score (mean ± SD)	*F*	*P*
Sex				47.767	0.000
	Male	1664 (42.0)	53.43 ± 12.697		
	Female	2299 (58.0)	56.15 ± 11.86		
Age				42.444	0.000
	<45 years old	3023 (76.3)	54.30 ± 11.61		
	≥45 years old	940 (23.7)	57.28 ± 14.02		
National				1.622	0.203
	Han	3799 (95.9)	55.06 ± 12.21		
	Non-Han	164 (4.1)	53.81 ± 14.09		
Marital status				0.706	0.494
	Married	2047 (51.7)	54.94 ± 12.37		
	Unmarried	1850 (46.7)	55.14 ± 12.14		
	Divorced or widowed	66 (1.7)	53.39 ± 14.18		
Residence area				1.714	0.191
	Rural	1188 (30.0)	55.17 ± 12.23		
	Urban	2775 (70.0)	54.62 ± 12.42		
Education level				33.996	0.000
	Non-higher education	1081 (27.3)	56.86 ± 13.49		
	Higher education	2882 (72.7)	54.31 ± 11.74		
Cancer cognition				16.282	0.000
	Unawareness	1991 (50.2)	55.79 ± 12.88		
	Awareness	1972 (49.8)	54.22 ± 11.61		
Monthly income				3.727	0.054
	≤5000 RMB	2189 (55.2)	55.35 ± 12.44		
	>5000 RMB	1774 (44.8)	54.59 ± 12.09		
Occupation				41.256	0.000
	Nonmedical staff	3070 (77.5)	55.68 ± 12.38		
	Medical staff	893 (22.5)	52.69 ± 11.69		
Personality				2.896	0.089
	Introvert	2152 (54.3)	55.31 ± 12.05		
	Extrovert	1811 (45.7)	54.65 ± 12.56		

ANOVA = analysis of variance, FOCS = fear of cancer scale.

### 3.2. FOCS pre-investigation review

A total of 7 experts were invited for consultation. In the first round of expert consultation, we reviewed and determined the meaning, grammar, and accuracy of each item, and reached a final agreement. In the second round of expert consultation, the I-CVIs of the 17 items were all >0.80, and the S-CVI was 0.926. All items were forward-scored, so a higher score indicated a greater level of cancer-related fear.

A total of 27 members of the general population were invited to participate in cognitive interviews. For each item, participants answered the same question: “Can you read and understand the content and significance of the item?” The I-FVIs of the 17 items were all >0.85, and the S-FVI was 0.936. Therefore, no revision of the item content was needed.

Through the pre-survey of 84 general population, it was found that the acceptance and completion rate of the questionnaire were 100%, and the average completion time was controlled within 20 minutes, which indicated that the questionnaire had good comprehensibility, acceptability and reality, and could be used for formal investigation.

### 3.3. FOCS validation

#### 3.3.1. Face and content validity

After expert consultation and cognitive interview, FOCS was considered to be able to effectively reflect the content and purpose of the study and to avoid “expectation bias” in participants’ responses. The I-FVIs of all 17 items of FOCS were from 0.861 to 0.948, and the S-FVI was 0.936, which indicated that it had good face validity.

The I-CVIs of all 17 items in the primary FOCS were from 0.821 to 0.964, and the S-CVI was 0.926. Each item was strongly correlated with the FOCS, and the Pearson correlation coefficients ranged from 0.450 to 0.675 (≥0.40, *P* < .01) (Table 2). Each item was strongly correlated with each subscale, and the Pearson correlation coefficients ranged from 0.648 to 0.818 (≥0.40, *P* < .01). Therefore, it was determined that the FOCS had good content validity.^[29]^

**Table 2:** Factor loadings distribution of each item in FOCS (n = 1610).

Items for FOCS	*r*	Common factors		
		F1	F2	F3
6. I fear cancer because it is very painful.	**0.667** *	**0.869**	0.143	0.047
5. I fear cancer because it could cause huge economic burden.	**0.647** *	**0.848**	0.164	-0.014
4. I fear cancer because it could endanger one’s life.	**0.675** *	**0.805**	0.235	0.032
7. I fear being diagnosed with cancer when I take medical checkups in hospitals.	**0.671** *	**0.779**	0.114	0.275
8. I fear cancer because it would make relatives and friends deeply sad.	**0.625** *	**0.777**	0.085	0.170
2. I fear my relatives and friends will have cancer.	**0.579** *	**0.738**	-0.057	0.273
1. I fear I will have cancer 1 day.	**0.594** *	**0.691**	0.023	0.323
3. I fear when I hear the word cancer.	**0.669** *	**0.638**	0.333	0.135
14. I fear associating with cancer patients.	**0.510** *	0.001	**0.842**	0.107
15. I fear cancer because it would cause isolation from other people.	**0.558** *	0.162	**0.764**	0.024
13. I fear going to hospital or ward specially for cancer patients and treatment.	**0.612** *	0.129	**0.764**	0.220
12. I fear seeing cancer patients.	**0.559** *	0.054	**0.743**	0.278
9. I fear cancer would be contagious.	**0.524** *	0.111	**0.673**	0.134
16. I fear cancer would be heritable.	**0.573** *	0.233	**0.560**	0.172
10. I suspect I have cancer when I hear other people are diagnosed with it.	**0.590** *	0.182	0.299	**0.757**
11. I fear my problem become cancer when I am sick.	**0.626** *	0.260	0.304	**0.707**
17. I would go to the hospital for cancer screening/exams when I hear people being diagnosed with it.	**0.450** *	0.141	0.137	**0.631**
Eigenvalues		4.995	3.631	1.961
Cumulative variance contribution rate (%)		29.382	50.742	62.277

The meaning of the bold values was that the factor loadings were >0.40.

FOCS = fear of cancer scale.

* *P* < .01.

#### 3.3.2. Convergent and discriminant validity

**Figure 1.**
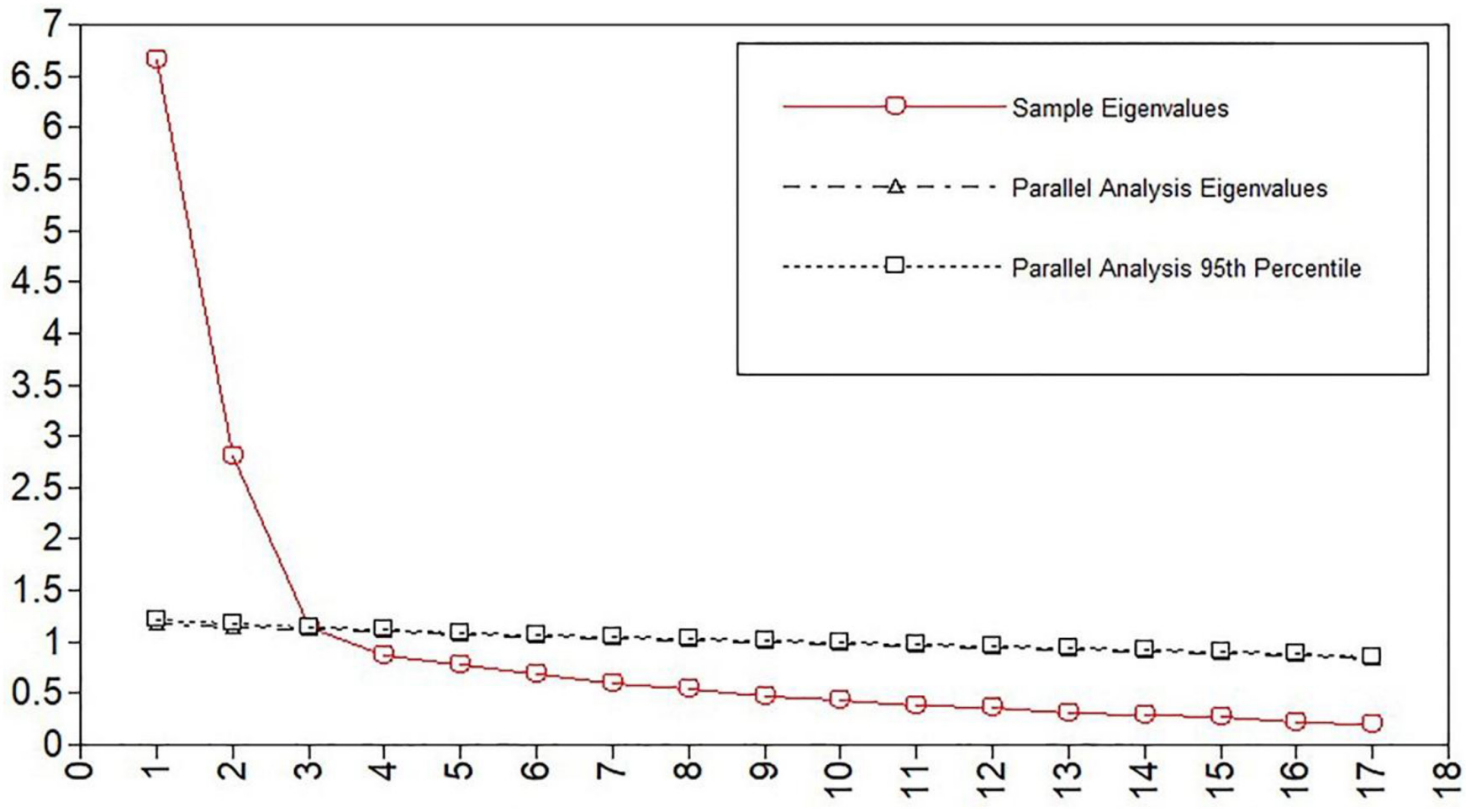
Eigenvalues for EFA. EFA = exploratory factor analysis.

A total of 1610 participants were recruited for EFA, and the results showed that the Kaiser–Meyer–Olkin coefficient of the FOCS was 0.912, and the Bartlett test was 14793.023 (*P* < .001), which indicated that the test was suitable for factor analysis.^[30]^ Parallel analysis results showed that the eigenvalues of 3 common factors were greater than the average eigenvalues of the random matrix (Fig. 1). Therefore, 3 common factors with characteristic roots >1 were extracted by principal component analysis, and the cumulative variance contribution rate was 62.277%. The factor loading of each item in its dimension was >0.40, so there was no need to remove any item from FOCS (Table 2). The average variance extracted and composite reliability of factor 1 were 0.506 and 0.880, factor 2 were 0.532 and 0.871, while factor 3 were 0.490 and 0.742, respectively. These results indicated that FOCS had good convergent and discriminant validity.

However, our study showed that FOCS was divided into 3 dimensions, which was different from prior research findings, which posited it into 2 dimensions.^[23]^ Therefore, we re-invited 6 participants with high FOCS scores to conduct a supplementary focus interview to determine whether the potential fear of cancer was real, as outlined below: What do you think are the main causes of cancer-related fears? Did you think that cancer in people around you would increase your fear of cancer? And did you need more medical checkups or consultations for those reasons? Four of the participants said their fear of cancer was largely due to the conventional wisdom that cancer was often considered incurable. Even if you didn’t have any symptoms, the fear of cancer increases with age, which was a kind of imprint on the brain that seemed difficult to erase.

Therefore, according to the expert consultation, focus interviews and research group discussions, it was finally decided to name Factor 1 direct fear, which mainly reflected the objective and genuine feelings that people were afraid that their safety would be threatened by cancer. Factor 2 was named indirect fear, which mainly reflected people’s awareness of subjective fear of cancer-related, which was easily spread by the surrounding environment and health knowledge. Factor 3 was named latent fear, which mainly reflected the phenomenon of cancer-related fear caused by the surrounding environment and subconscious changes in the brain, often inadvertently generated, but not drastic, which was obviously distinguished from indirect fear. For example, the general population experiences self-perceived emotional distress of learning that someone else had been diagnosed with a malignant tumor, but was not affected by the actual disease themselves. Therefore, we ultimately adjusted FOCS into 3 dimensions: direct fear, indirect fear, and latent fear.

#### 3.3.3. Construct validity

**Figure 2.**
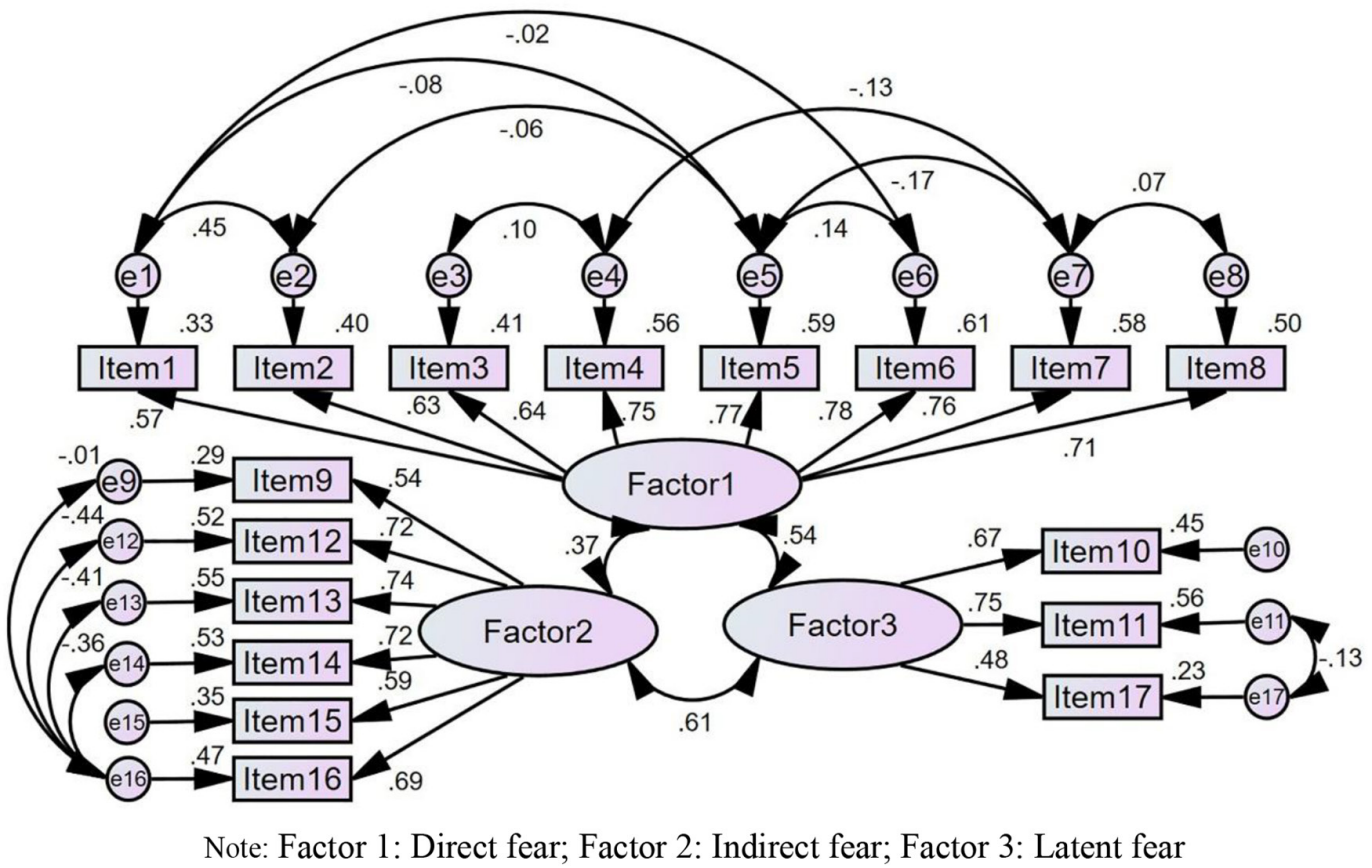
The three-factor correlation model of FOCS. Factor 1: Direct fear; Factor 2: Indirect fear; Factor 3: Latent fear. FOCS = fear of cancer scale.

According to the results of EFA, 2 theoretical models were constructed by structural equation modeling: a three-factor independent model and a three-factor correlation model. A total of 2353 participants were recruited for CFA, and the results showed that the fitting degree of the three-factor correlation model was better than that of the three-factor independent model (Table 3). The chi-square degree of freedom ratio was 5.246, >5, which indicated that the adaptability of the model was average. However, due to the susceptibility of chi-square tests to sample size, chi-square tests often fail when the sample size is large. Therefore, the model fitting validity of this study mainly referred to other indices. Except for the chi-square degree of freedom ratio, the results of all evaluation index were greater than the minimum threshold, which indicated that the three-factor correlation model of FOCS had good structural validity (Figure 2 and Table 3).^[31,32]^

**Table 3:** Fitting index of CFA for FOCS (n = 2353).

Structural model	*χ^2^* */df*	RMSEA	GFI	AGFI	CFI	NFI	IFI	TLI
Three-factor independent model	12.528	0.070	0.930	0.908	0.912	0.905	0.912	0.897
Three-factor correlation model	5.246	0.042	0.974	0.961	0.972	0.965	0.972	0.962
Threshold	<5.00	<0.05	>0.90	>0.90	>0.90	>0.90	>0.90	>0.90

AGFI = adjusted goodness of fit index, CFI = comparative fit index, GFI = goodness of fit index, IFI = incremental fit index, NFI = normed fit index, RMSEA = root mean square error of approximate, TLI = tucker-Lewis index, *χ^2^/df* = chi-square degree of freedom ratio.

#### 3.3.4. Reliability

The Cronbach alpha of the final FOCS was 0.885, and the Spearman-Brown coefficient was 0.674. The Cronbach alpha values of the 3 subscales were 0.898, 0.820 and 0.662, and the Spearman-Brown coefficients were 0.862, 0.811 and 0.548, respectively. These results indicated that FOCS generally had good internal and split-half reliability. However, the split-half reliability of the latent fear subscale was average, which might be related to its small number of items.^[33]^ One month after the first questionnaire, a total of 34 participants of the general population were invited for a second questionnaire to verify the test-retest reliability of the scale. The test-retest results showed that the intra-class correlation coefficients of FOCS and 3 subscales were 0.840, 0.746, 0.792 and 0.833, which indicated that FOCS had good test-retest reliability.^[34]^

#### 3.3.5. Differential item function

Ordered logistic regression was used to analyze DIF of each item in the scale, and it was found that the analysis results of items 4, 9, 13, 15, and 16 had statistically significant (*P* < .01), which indicated that the above 5 items had DIF for FOCS in different sex groups (Table 4).

**Table 4:** DIF analysis of ordered logistic regression in different sex populations.

Items	Regression coefficient	*P*
1. I fear I will have cancer 1 day.	0.126	.048
2. I fear my relatives and friends will have cancer.	0.067	.318
3. I fear when I hear the word cancer.	−0.148	.017
4. I fear cancer because it could endanger one’s life.	0.195	.004
5. I fear cancer because it could cause huge economic burden.	0.074	.286
6. I fear cancer because it is very painful.	−0.143	.045
7. I fear being diagnosed with cancer when I take medical checkups in hospitals.	−0.097	.153
8. I fear cancer because it would make relatives and friends deeply sad.	0.016	.814
9. I fear cancer would be contagious.	0.166	.007
10. I suspect I have cancer when I hear other people are diagnosed with it.	0.142	.028
11. I fear my problem become cancer when I am sick.	−0.010	.880
12. I fear seeing cancer patients.	−0.118	.063
13. I fear going to hospital or ward specially for cancer patients and treatment.	−0.242	.000
14. I fear associating with cancer patients.	0.050	.439
15. I fear cancer because it would cause isolation from other people.	0.281	.000
16. I fear cancer would be heritable.	−0.279	.000
17. I would go to hospital for cancer screening/exams when I hear people being diagnosed with it.	−0.085	.160

DIF = differential item function.

#### 3.3.6. Applicability

A total of 4342 FOCS questionnaires were distributed, and 3963 valid questionnaires were recovered (the effective completion rate was 91.27%≥90%). The average completion time of the FOCS was 298.13 ± 191.484 (range: 74 to 1688) seconds (≤900 seconds). Therefore, it was determined that the FOCS had good applicability.^[35]^

### 3.4. Risk factors of FOCS among the general population

After the above scale adjustment and verification, it was proven that the adjusted FOCS could be applied in the general population. The FOCS scores of 3963 participants were 17~85, with *M (P*_25_, *P*_75_) of 55 (48, 63), which proved that the general population generally had a strong fear of cancer-related.

One-way analysis of variance indicated statistically significant differences in sex, age, occupation, education level, and cancer cognition (*P* < .01) (Table 1). The FOCS score was used as the dependent variable, and the above 5 variables with statistical significance were used as independent variables. Multiple stepwise linear regression analysis revealed that sex (male or female), age (<45 years old or ≥45 years old), occupation (nonmedical staff or medical staff), and education level (senior high school and below or college and above) were independent risk factors for general population cancer-related fear (*P* < .01), and the variance inflation factor of the 4 independent variables in the regression model was very close to 1 (Table 5).

**Table 5:** Independent risk factors for FOCS.

Variable	Regression coefficient	Standard error	Normalized regression coefficient	*t*	*P*	*VIF*
Sex	3.320	0.392	0.133	8.469	.000	1.022
Age	2.689	0.465	0.093	5.784	.000	1.068
Occupation	−1.368	0.234	-0.093	−5.854	.000	1.040
Education level	−1.857	0.451	-0.067	−4.121	.000	1.100

Assignment: age: 1 = male, 2 = female; sex: 1 was <45 years old, 2 was ≥45 years old; occupation: 1 = nonmedical staff, 2 = medical staff; education level: 1 = senior high school and below, 2 = college and above.

FOCS = fear of cancer scale.

## 4. Discussion

Our study showed that the general population had a strong fear of cancer, which may be the comprehensive embodiment of a variety of reasons, including direct, indirect and potential. People not only had objective and authentic fears about cancer, but also had subjective and potential fears of being susceptible to infection, and the latent fear was often inadvertently generated and usually not very drastic, but difficult to eliminate. Consequently, we applied the FOCS into entire Chinese general population, and further tested its reliability, validity and applicability. After EFA, it was found that FOCS of 17 items were divided into 3 dimensions, which was different from previous studies.^[23]^ Therefore, we invited 6 participants for focus interviews and made it clear that the potential fear of cancer in the general population was real. Finally, we adjusted FOCS into 3 subscales: direct fear (8 items), indirect fear (6 items), and latent fear (3 items).

After the classical test of the scale, it was found that the 3 dimensions correlation of FOCS had good face and content validity (I-FVIs > 0.85, I-CVIs > 0.80, Pearson correlation coefficients > 0.30 [*P* < .01]), convergent and discriminant validity (Kaiser–Meyer–Olkin  = 0.912, Bartlett test = 14793.023 [*P* < .001], cumulative variance contribution rate = 62.277%, and all factor loadings > 0.40), construct validity (root mean square error of approximate = 0.042, goodness of fit index = 0.974, AGFI = 0.961, comparative fit index = 0.972, normed fit index = 0.965, incremental fit index = 0.972, tucker-lewis index = 0.962), reliability (Cronbach α coefficient = 0.885, Spearman-Brown coefficient = 0.674, intra-class correlation coefficient = 0.840), and applicability (average completion time was 298.13 ± 191.484 seconds, effective completion rate was 91.27%). Therefore, adjusting FOCS to 3 dimensions correlation may be more effective and reliable in assessing the psychological fear characteristics of the objective reality and subjective potential of malignant tumors among the general population.

Compared with CAM, the adjusted FOCS had greater simplicity, reliability, and specificity. The reliability of the FOCS was greater than that of the CAM (Cronbach's α coefficient is 0.77).^[20]^ The CAM was a universal scale for health knowledge awareness, which could be used only to preliminarily evaluate people’s cognition level of cancer-related knowledge, and couldn’t be used to estimate psychological fear associated with cancer, so it still lacked specificity. In addition, compared with the 55-item CAM, the 17-item FOCS had obvious advantages in terms of applicability and convenience, which indicated that it was easier for the general population to accept and understand, and was conducive to its popularization and application. Compared with CBCFS, the FOCS had a wider range of applications. The CBCFS could be used only to assess the fear of breast cancer in healthy women undergoing mammography.^[17]^ Although the FOCS was developed based on college students, our study showed that it was applicable to all of the general population. Therefore, the adjusted FOCS may be a more promising early psychological counseling and screening tool for cancer-related fear in the general population.

Notably, our study found that FOCS scores of 3963 participants were 17 to 85, with *M (P*_25_, *P*_75_) being 55 (48, 63), which indicated that the general population had a strong fear of cancer-related. Based on our findings, we recommend that participants score <48 for mild cancer-related fear, 49 to 63 for moderate cancer-related fear, and >64 for severe cancer-related fear. In the stereotype of the Chinese people, cancer has always been regarded as an unpredictable and difficult to overcome malignant disease, which has led people to widely experience the psychological barrier of “cancer peculiar horror.” The public’s fear of cancer is easily influenced by unscientific rumors, and people may even mistakenly believe that cancer is an “ominous” sign, and thus deliberately avoid cancer patients.^[36]^ In addition, the fact that cancer treatment can cause patients to experience both psychological and physical pain, which leads the public to believe that once they have cancer, it is difficult for them to escape from the cancer “haze,” even cancer patients with low malignancy and good prognosis will eventually lead to death.^[37]^ Therefore, we suggest that FOCS can be used for cancer screening and health education programs in communities and outpatient clinics to identify individuals with moderate to severe fear and take necessary interventions to alleviate cancer-related psychological burden.

Interestingly, we found that education level, occupation, sex and age were independent risk factors for general population fear of cancer (*P* < .01). The public with lower education levels had greater fear of cancer. Education level is a prerequisite for one’s career and income, which plays an important role in following healthy lifestyle habits and accessing healthcare.^[38]^ With the promotion of quality education in China, people’s cognition of cancer has gradually improved, which has significantly relieved the public’s fear of cancer-related and reduced the risk of cancer-related death.^[39]^The fear of cancer was stronger in women and middle-aged and elderly people, which might be closely related to the age of cancer onset, and the decline in personal physical fitness and psychological state. As the function of tissues and organs decreases, middle-aged and elderly people are prone to varying degrees of physiological and psychological changes. The emergence of cancer warning symptoms has intensified their psychological burden, and frequent tumor screening undoubtedly exacerbates their fear of cancer-related. This phenomenon may be more pronounced in women and even lead to severe “menopausal cancer phobia.”^[12,40]^ In addition, healthcare workers had a significantly lower fear of cancer than did other populations. In China, because of the lack of health knowledge, nonhealthcare workers are easily affected by adverse information related to cancer. Most people taboo cancer because of fear, discrediting cancer pathogenesis factors and diagnosis and treatment knowledge, which may be associated with personal misconduct, leading to many psychological “halo effects.”

In conclusion, occupation and age determine objective risks, while education level determines subjective risk perception. Age, gender, occupation, and education level determine social roles and economic status, which collectively affect the ability to access medical resources, health literacy, and strategies for coping with cancer fear.^[41,42]^ Therefore, strengthening health education to enhance general population's cognition of cancer, eliminating people’s “halo effect” on cancer, and changing people’s inherent thinking of “cancer peculiar horrors” are the keys to reducing cancer-related fear. Second, it is particularly important to create a positive, upward and healthy environment in key living areas such as hospitals, communities and schools to eliminate the “haze” of public cancer psychology. In addition, social support and sympathy are equally important. By building a harmonious, open, and inclusive social atmosphere, we encourage everyone to accept and respect cancer patients and reduce unequal, negative, and discriminatory speech and behavior.

However, due to the lack of recognized reference standards, the criterion-related validity of the FOCS was not tested in this study. In addition, our research showed that the internal reliability of the latent fear subscale was average, which might be related to its small number of items. And item 4, 9, 13, 15, and 16 had significant gender-specific DIF, so we believe that the above items in FOCS need further revision and validation in the future. The random sampling and snowball sampling method were adopted in this study, which resulted in an imbalance of sample proportions to different degrees (for example, the ratio of individuals ≥45 years old to those < 45 years old was 1:3.22). Because of the limited sample size, our proposed classification criteria for fear degree were used only for preliminary reference. Due to the limitations of the cross-sectional study design, it is impossible to establish a clear causal relationship. Furthermore, as this study mainly focused on Chinese people, the applicability of its results in other cultural contexts remains to be verified. Future research should include a larger sample size to analyze the correlation between cancer-related fear and other negative psychological factors (anxiety, depression, etc), and track the changes in cancer fears over time through longitudinal studies, as well as the long-term impact of health education interventions on reducing cancer fears. In addition, with the widespread application of genomics, multi-omics techniques, and machine learning methods in biomedical research, the future can explore the combination of these advanced technologies with psychological assessment tools to develop more accurate cancer fear prediction and intervention models.

## 5. Conclusion

Our study demonstrated that the general population commonly experience significant fear of cancer, and the adjusted FOCS was proven to be effective and reliable for early identification and assessment of these problems. This tool may serve as a foundation for early psychological counseling and screening for cancer fear among the general population, particularly in China. Timely and effective intervention measures targeting risk factors will aid in the prevention and alleviation of the general population cancer-related fear.

## Acknowledgments

We would like to extend our heartfelt gratitude to the noncancer population for their participation.

## Author contributions

**Investigation:** Li Feng, Yu Chunmei, Wan Fangyun, Wang Wenxing, Wang Xuexing, Zhang Jian, Xu Jun, Gan Lin.

**Validation:** Li Feng, Yu Chunmei.

**Writing – original draft:** Li Feng, Yu Chunmei, Wan Fangyun.

**Data curation:** Yu Chunmei.

**Formal analysis:** Wan Fangyun.

**Conceptualization:** Liu Zhijin.

**Funding acquisition:** Liu Zhijin.

**Project administration:** Liu Zhijin.

**Supervision:** Liu Zhijin.

**Writing – Review & Editing:** Liu Zhijin.
